# DYT1 Knock-In Mice Are Not Sensitized against Mitochondrial Complex-II Inhibition

**DOI:** 10.1371/journal.pone.0042644

**Published:** 2012-08-03

**Authors:** Nicole Bode, Cory Massey, Pedro Gonzalez-Alegre

**Affiliations:** 1 Department of Neurology, Roy J and Lucille Carver College of Medicine at the University of Iowa, Iowa City, Iowa, United States of America; 2 Graduate Program in Neuroscience, the University of Iowa, Iowa City, Iowa, United States of America; Chiba University Center for Forensic Mental Health, Japan

## Abstract

DYT1 is caused by a partly penetrant dominant mutation in *TOR1A* that leads to a glutamic acid deletion (ΔE) in torsinA. Identifying environmental factors that modulate disease pathogenesis and penetrance could help design therapeutic strategies for dystonia. Several cell-based studies suggest that expression of torsinA(ΔE) increases the susceptibility of neuronal cells to challenges to their oxidative/energy metabolism. Based on those reports, we hypothesized that mice expressing torsinA(ΔE) would be more susceptible than control littermates to the effects of oxidative stress and ATP deficits caused by disruption of the mitochondrial respiratory chain in neurons. To test this hypothesis, we administered 20 or 50 mg/kg/day of the irreversible complex-II inhibitor 3-nitropropionic acid (3-NP) intraperitoneally for 15 consecutive days to young heterozygote DYT1 knock-in (KI) mice and wild type littermates. Repeated phenotypic assessments were performed at baseline, during and after the injections. Animals were then sacrificed and their brains processed for protein analysis. The administration of 20 mg/kg 3-NP led to increased levels of torsinA in the striatum, the main target of 3-NP, but did not cause motor dysfunction in DYT1 KI or control mice. The administration of 50 mg/kg/day of 3-NP caused the death of ∼40% of wild type animals. Interestingly, DYT1 KI animals showed significantly reduced mortality. Surviving animals exhibited abnormal motor behavior during and right after the injection period, but recovered by 4 weeks postinjection independent of genotype. In contrast to the findings reported in cultured cells, these studies suggest the DYT1 mutation does not sensitize central neurons against the toxic effects of oxidative stress and energy deficits.

## Introduction

Dystonia is a movement disorder characterized by twisting involuntary movements that lead to abnormal postures [1]. Both genetic and environmental factors play a role in dystonia pathogenesis. However, how they interact remains unknown. Answering this question could help us gain a better understanding of the neurobiological process underlying both inherited and sporadic forms of this heterogeneous condition. The identification of causative genes in some forms of inherited dystonia has led to the generation of animal models that can be used to explore the neurobiological bases of this syndrome [2], including gene-environment interactions.

DYT1, the most common inherited, early-onset, generalized dystonia [3], is caused by dominant mutations in the gene *TOR1A* which encodes the protein torsinA [4]. Most patients with DYT1 present in the first two decades of life with dystonia affecting an extremity that subsequently generalizes, causing significant disability [5]. An interesting feature of the DYT1-causing mutation is its reduced clinical penetrance. Only about a third of mutation carriers develop symptoms. Although a single nucleotide polymorphism in the *TOR1A* gene has been found to modulate disease penetrance, this only accounts for a small effect [6]. The identification of environmental modifiers of disease pathogenesis and penetrance is a critical goal on dystonia research, as it could help us design preventive or therapeutic strategies. Recent studies of the disease-causing protein have identified different biological pathways influenced by torsinA function [7], [8]. If these pathways are susceptible to environmental influences, they could be at the center of a gene-environment interaction in the pathogenesis of dystonia.

TorsinA is a widely expressed AAA (ATPases Associated with diverse cellular Activities) endoplasmic reticulum (ER) glycoprotein [4]. How torsinA dysfunction causes dystonia is unknown. Interestingly, multiple reports suggest a link between torsinA function, energy metabolism and redox biology. First, the four members of the mammalian family of torsin proteins (torsinA, torsinB, torsin2A and torsin3A) reside in the highly oxidizing ER environment and have highly conserved cysteines [9]. Second, torsinA forms intramolecular disulfide bonds through essential cysteines that regulate its ability to bind ATP/ADP and protein substrates [9], [10]. Third, H_2_O_2_ modifies the subcellular localization and electrophoretic properties of torsinA in cultured cells [11]. Fourth, torsinA overexpression influences levels of proteins implicated in energy metabolism and redox control [12]. Fifth, torsinA expression protects cultured cells and dopaminergic neurons in *c. elegans* against oxidative agents [13], [14], [15], [16]. Finally, torsinA is upregulated in the rat brain upon ischemia [17] and exposure to MPTP [18], a complex I inhibitor that causes an energy deficit and the buildup of free radicals. Collectively, these reports suggest that challenges to the neuronal energy/redox states could be a trigger for the pathogenic cascade in DYT1.

Based on this information, we hypothesized that the mammalian DYT1 brain is sensitized to the effects of energy depletion and oxidative stress caused by disruption of the mitochondrial respiratory chain, which would trigger the disease phenotype. To test this hypothesis, we administered the irreversible complex-II inhibitor 3-nitroproprionic acid (3-NP), a toxin known to cause dystonia in rodents, primates and humans [19], to DYT1 *knockin* (KI) mice.

## Materials and Methods

### Ethics Statement

This study was carried out in strict accordance with the recommendations in the Guide for the Care and Use of Laboratory Animals of the National Institutes of Health. The protocol was approved by the Institutional Animal Care and Use Committee at the University of Iowa (Animal Protocol #0906127). All efforts were made to minimize suffering. During the period when animals received 3-NP or saline, they were monitored daily by the investigators and the animal care unit personnel to evaluate for physiological indicators of discomfort that included weight loss, food and water intake, activity and presence of paw cyanosis. If necessary, to minimize suffering, animals were sacrificed with 1000 mg/kg xylazine and 100 mg/kg ketamine intraperitoneally. Despite these measures, death as a consequence of 3-NP was not predictable, and animals that died were undistinguishable from littermates. No animals had to be sacrificed.

### Animals

For these studies we used heterozygous DYT1 KI mice, generated by Dauer and colleagues [20]. These animals do not exhibit any motor dysfunction or abnormal brain morphology. However, functional neuroimaging identified abnormal patterns also found in humans carriers of the DYT1 mutation [21]. Therefore, this is a model of non-manifesting DYT1 mutation carriers, and will be useful to evaluate candidate triggers of phenotypical penetrance. Mice were maintained on a 129/SvEv background. Mice were housed in controlled temperature rooms with 12 hr light and dark cycles. Food and water were provided ad libitum. Genotype was assessed by PCR. All experimental protocols were approved by the University of Iowa Animal Care and Use Committee.

### 3-nitropropionic acid treatment regimens

**Figure 1:**
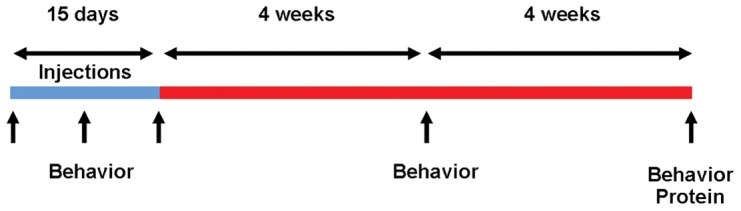
Experimental design. Mice were 6–8 weeks of age at the beginning of the injections. Behavioral analyses include spontaneous locomotion and performance on the rotarod. Blue: acute toxicity period. Red: recovery period.

While acute administration of high doses of 3-NP cause significant striatal damage, motor dysfunction and animal death, the subacute/chronic administration of low doses of this toxin causes transient weight loss and motor dysfunction that is fully reversible [22]. Because DYT1 is a functional, non-degenerative disorder, we pursued regimens with administration of low doses of 3-NP for 15 days. Susceptibility to 3-NP toxicity is animal species, strain and age dependent. Prior studies with the mouse strain used here suggest the doses of 75 mg/kg/day would cause significant morbidity and mortality [23]. We elected to use two different lower doses for our experiments. Six to eight week-old DYT1 KI mice and control littermates were used in all experiments. A first group of animals (n = 10/group, 5 males and 5 females) received 3-NP (20 mg/kg/day) or an equivalent volume of 0.9% saline administered intraperitoneally (IP) daily for 15 consecutive days. A second group of mice received a chronic “high-dose” of 3-NP (50 mg/kg/day) or saline control following the same protocol. Due to the mortality observed with this regimen, we used a higher number of animals per experimental group: WT-Saline (n = 10, 4 males/6 females), DYT1 KI-Saline (n = 14, 6 males/8 females), WT-3-NP (n = 15, 8 males/7 females), DYT1 KI-3-NP (n = 16, 8 males/8 females). The experimental protocol is illustrated in Figure 1.

### Behavioral analysis

Weights were monitored daily during the injection period and weekly thereafter. Spontaneous locomotor activity was evaluated as described [24] using a an automated tracking system (Viewpoint, Videotrak, Lyon, France) at baseline, at day 8 of the injections, at the conclusion of the injection period and 4 and 8 weeks later. Briefly, after acclimation, distance traveled, speed and transitions between different speeds were recorded for 4 mice simultaneously in an open field arena with 4 separate 25 cm×25 cm fields. We collected and analyzed data in 5 min bins for 30 min. Performance on the accelerating rotarod (model 47600 Ugo Basile, Italy) was recorded following our published protocol [24]. Mice were acclimated to the rotating rod for 5 min at an accelerating rate from 4 rpm to 16 rpm. They were then tested for 3 days with 3 trials per day on a rotarod set to accelerate from 4 rpm to 40 rpm over a period of 5 min. Latency to fall was recorded on each day with a maximum cutoff at 500 sec, and the average performance recorded at baseline, at the conclusion of the injection period and 4 and 8 weeks later. These tests of motor function were selected as they have been previously reported to be slightly abnormal in DYT1 mouse models [25], [26], [27]

### Protein analyses

Mice were perfused transcardially with ice cold saline, their brain extracted, the striata manually dissected, snap frozen with liquid nitrogen and stored in an ultra-freezer until used. RIPA buffer with Complete Mini Protease Inhibitor Cocktail (PI) (Roche) was used to collect protein lysates as described [24]. Lysates were diluted in Laemmli buffer previous to SDS-PAGE followed by Western blot analysis for torsinA (ab34540 antibody, Abcam, Cambridge, MA) and α-tubulin (T5168 antibody, Sigma, St. Louis, MO). For quantification of western blot signal we followed our reported protocol using ImageJ software, normalizing to α-tubulin as a loading control [24].

### Statistical analysis

GraphPad Prism 5 (GraphPad Software, Inc., La Jolla, CA) was used for statistical analyses. The Gehan-Breslow-Wilcoxon test was used for survival analysis. Repeated measures two-way ANOVA was used to analyze the effect of treatment on the different behavioral measures analyzed over time as described in the results section and figure legends.

## Results

Based on cell-based studies showing a higher susceptibility of cells expressing torsinA(ΔE) to oxidative stressors, we hypothesized that chronic administration of low doses of the complex-II inhibitor 3-NP would trigger a motor phenotype in DYT1 KI mice but not in control littermates. To test this hypothesis, we first administered a low dose regimen of 3-NP (20 mg/kg/day IP for 15 days or saline control). Both DYT1 and WT mice tolerated well this 3-NP regimen, with no effect on survival, weight or motor performance when compared to saline-treated mice (not shown). However, protein analysis by western blotting showed higher levels of torsinA after treatment with 3-NP, more evident for WT than DYT1 mice (shown later with the 50 mg/kg/day group). Thus, despite having a detectable effect on the rodent striatum, subclinical inhibition of the mitochondrial respiratory chain did not trigger motor dysfunction in DYT1 KI mice, arguing against the presence of significantly enhanced sensitivity to this toxin as a result of the DYT1 mutation.

**Figure 2:**
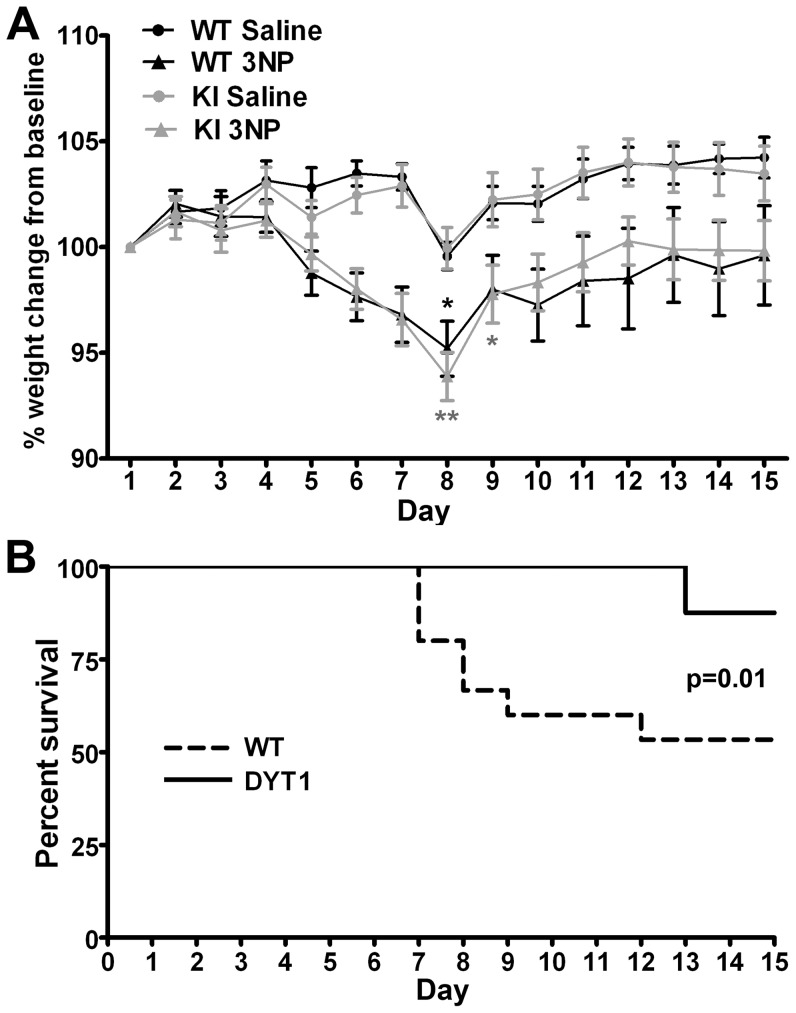
DYT1 KI mice are resistant to death caused by 3-NP. (A) Change in weight during the injection period expressed as a percentage of the initial weight. Two way ANOVA for repeated measures demonstrates a significant interaction between time and experimental group (*F*[42,630] = 3.34; p<0.0001). Post-test Bonferroni was done using the WT Saline group as a reference (*p<0.05; **p<0.01). (B) Kaplan Meyer survival curve for DYT1 and control mice demonstrates statistically significant differences in mortality upon treatment with 3-NP between both genotypes. The Gehan-Breslow-Wilcoxon test was used for statistical analysis. All animals that received saline survived. There was no mortality beyond the injection period.

Mice are relatively resistant to the toxic effects of 3-NP, and this is influenced by genetic background [23]. Therefore, it is possible that 20 mg/kg/day of 3-NP was too low to trigger a phenotype even in susceptible mice. For that reason, we used a higher dose regimen (50 mg/kg/day). Mice receiving this dose exhibited significant weight loss during the injection period when compared to saline-treated controls, independent of genotype (Figure 2A). The peak in weight loss occurred at day 8 as previously reported [28]. There were no deaths among animals receiving saline. However, 3-NP caused significant mortality in WT mice receiving 3-NP. Unexpectedly, DYT1 KI mice were protected from mortality caused by 3-NP (Figure 2B).

**Figure 3:**
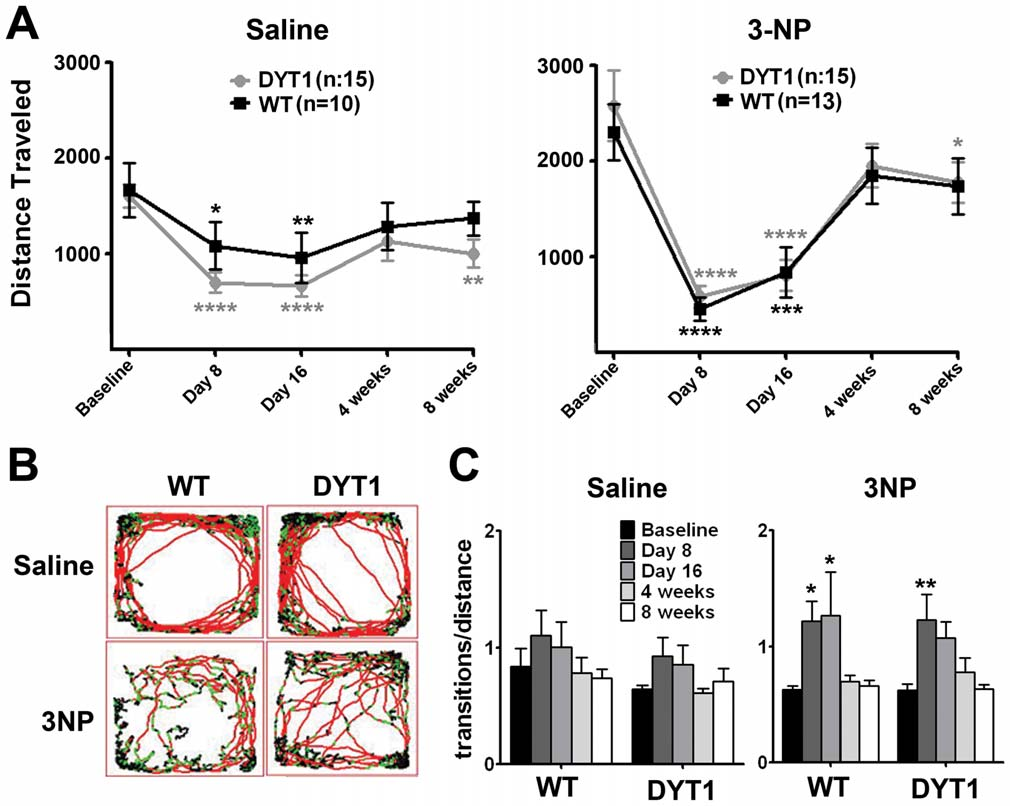
The effects of 3-NP on motor behavior are not influenced by the DYT1 genotype. (A) Distance traveled over a 30 minutes period at baseline, at day 8 of the injections (“crisis”), at the end of the IP injections and 4 and 8 weeks later. Repeated measures two-way ANOVA showed no interaction between genotype and time point for animals receiving saline or 3-NP and no effect of genotype but a significant effect of time in both the saline (*F*[4,84] = 11.83; p<0.0001) and 3-NP (*F*[4,92] = 29.42; p<0.0001) groups. Post-hoc Bonferroni analysis was performed comparing each time point to the baseline value, with significance shown in the graph (*p<0.05, **p<0.01, ***p<0.001, ****p<0.0001) (B) The pattern of the movement was affected by the administration of 3-NP, as mice developed hindlimb dysfunction. Shown are representative plots of locomotion for individual animals at day 16. Black line denotes no movement, green line slow movements and red line fast movements. (C) Analysis of the number of transitions between speeds per distance, indicating erratic movements, indicates a significant worsening of the gait pattern in the immediate post-injection period for the 3-NP group. Repeated measures ANOVA demonstrated no interaction between genotype and performance over time and no effect of genotype in the saline and 3-NP groups. There was a significant effect of time in the saline (*F*[4,84] = 3.44; p = 0.01) and 3-NP (*F*[4,92] = 9.38; p<0.0001) groups. Post-hoc Bonferroni analysis comparing each value to baseline demonstrates a significant increment in the number of transitions at days 8 and 16 for the 3-NP but not the saline group (*p<0.05, **p<0.01).

We next asked if the DYT1 mutation influences the development of 3-NP–induced motor dysfunction. We evaluated spontaneous locomotion at baseline, at injection day 8 (peak of weight loss), right after completion of the injection period and 4 and 8 weeks later. For treatment (saline or 3-NP), we analyzed the performance of each genotype over time. All treatment groups showed a reduction in total distance traveled at days 8 and 16 (right after the injections), perhaps due to the stress of the daily injections, with partial recovery 4 and 8 weeks later. However, repeated measures two-way ANOVA did not show any influence of genotype (DYT1 KI versus WT) on this outcome (Figure 3A). While the reduction on distance traveled at days 8 and 16 seem to be more pronounced for animals receiving 3-NP than saline, they also differed in their different baseline values. To further delineate the effects of 3-NP on locomotion, we evaluated the locomotor plots of all animals. 3-NP-treated animals showed an abnormal and erratic locomotion pattern, with frequent pauses and transitions between speeds (Figure 3B). To objectively quantify this abnormality, we determined the number of times each animal transitioned between different speeds of movement per distance traveled. Repeated measures two way ANOVA showed that this value changed over time without influence of genotype (WT and DYT1) (Figure 3C). Post-hoc Bonferroni analysis showed that the increment in the number of transitions during the injection period (day 8) was significant in animals receiving 3-NP but not saline, indicating a toxic effect of this toxin that recovered 4 weeks later (Figure 3C). Overall, the evaluation of spontaneous locomotion detected toxic effects of 3-NP that were reversible in a 4 week period. However, we found no differences between DYT1 KI and control mice in the presence of motor dysfunction or its recovery.

**Figure 4:**
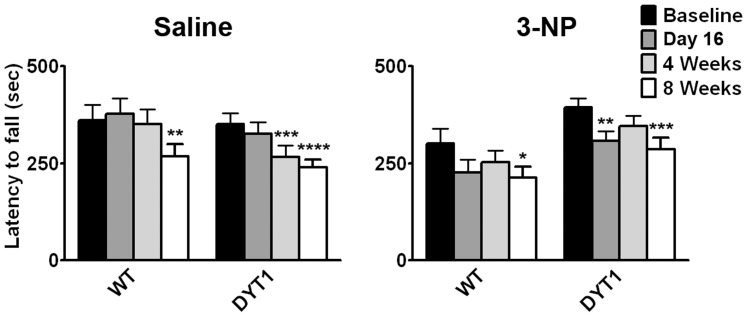
DYT1 genotype does not influence performance in the rotarod after the administration of 3-NP. The latency to fall from the accelerating rotarod was measured 3 times daily for 3 consecutive days at the different time-points indicated. The average value for each time point was used for analysis. Two-way ANOVA for repeated measures showed no interaction between genotype and time point for the saline or 3-NP groups and no effect of genotype. Time influenced performance in both the saline (*F*[3,66] = 16.72; p<0.0001) and 3-NP (*F*[3,69] = 8.81; p<0.0001) groups. Post-test Bonferroni comparisons to performance at baseline were done (*p<0.05, **p<0.01, ***p<0.001, ****p<0.0001).

Because performance on the rotarod is abnormal in some mouse models of DYT1 and is also affected by the administration of 3-NP, we evaluated this test of motor coordination. Performance in both the saline or 3-NP groups changed over time, but genotype did not influence this outcome (Figure 4). Overall, and in agreement with the locomotion data, these results argue against a higher sensitivity of DYT1 KI mice to 3-NP.

**Figure 5:**
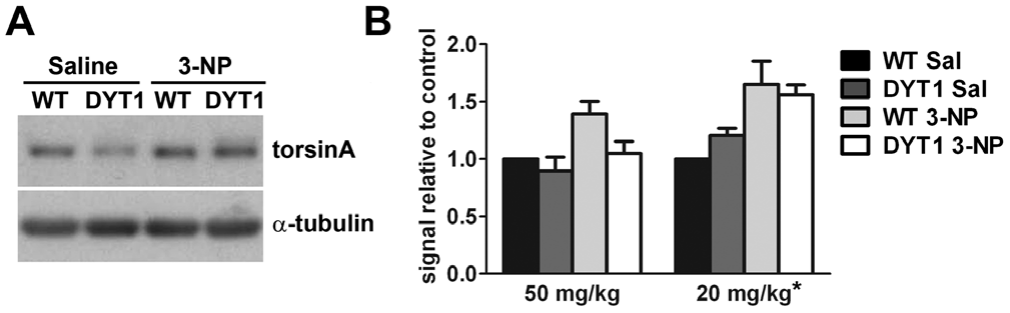
Levels of torsinA in the striatum are slightly increased upon treatment with 3-NP. (A) Representative western blot showing torsinA expression in striatal lysates of animals receiving 50 mg/kg/day of 3-NP or saline controls. (B) Quantification of torsinA expression as described in the text for animals that received 50 or 20 mg/kg/day of 3-NP (N: 5–7/group). ANOVA showed a non-significant trend in the 50 mg/kg/day group (p = 0.07) and significant differences in the 20 mg/kg/day group (*p = 0.01).

Eight weeks after the last injection, mice were sacrificed and their brains extracted for protein analysis. Levels of torsinA were evaluated by western blotting of striatal lysates. Similar to animals that received the low dose regimen, animals receiving 50 mg/kg/day of 3-NP showed a non-significant trend towards higher levels of torsinA when compared to saline-treated controls (Figure 5).

## Discussion

Cell-based studies have shown that overexpression of torsinA(ΔE) or downregulation of endogenous torsinA(wt) renders neural cells susceptible to oxidative stress. Based on these and other findings, we hypothesized that DYT1 KI mice would be more sensitive than control mice to the toxic effects of 3-NP. Our initial goal was to pursue additional studies to explore the mechanistic bases of such phenomenon. However, our experiments strongly argue against this hypothesis and, therefore, mechanistic studies were not performed. In fact, our findings suggest that the DYT1 mutation protects mice from death caused by 3-NP. In agreement with previous reports, we found that torsinA is upregulated in the mammalian brain under acquired neuronal metabolic/redox compromise. These results argue against a critical role of oxidative stress/ATP deficits as a “second hit” that triggers clinical penetrance in DYT1 mutation carriers.

We embarked on this study based on reports from different laboratories obtained in cultured cells and nematodes that showed a protective effect of torsinA against agents that cause oxidative stress [13], [14], [15], [16]. Why did we obtain the opposite result? First, instead of H_2_O_2_, we employed an agent that causes in addition to oxidative stress, energy depletion and excitotoxicity [19]. Second, we pursued a chronic toxic regimen that causes reversible motor dysfunction rather than chronic deficits due to severe striatal damage. Third, developmental compensatory changes could occur in the DYT1 KI mouse brain, but not upon acute overexpression or silencing of torsinA, that modify the neuronal response to this toxic insult. Fourth, recent studies have shown that torsinB, which is expressed in non-neuronal cells *in vivo* and the cells lines used for the above referenced experiments, is functionally redundant with torsinA [29], [30]. Finally, there is a fundamental difference between cell-based overexpression and this mouse model. Cultured cells overexpressing very high levels of the torsinA(ΔE) transgene contain two normal torsinA alleles expressed at endogenous levels. However, in DYT1 KI mice, there are a normal and a mutated allele, both expressed at physiological levels. This difference might very well play a role in the divergent results observed in both systems, for instance, if the DYT1 mutation causes a loss of function. These and other potential reasons for the discrepancy observed between cultured cells and animal models highlight the importance of performing confirmatory experiments in mammalian models *in vivo* in dystonia research.

Our data argues against defects in oxidative phosphorylation as a “second hit” that triggers dystonia in DYT1 mutation carriers. However, the unexpected finding of reduced mortality in DYT1 KI mice treated with toxic doses of 3-NP is still consistent with a link between torsinA function and this biological pathway. This is also supported by the upregulation of torsinA expression in the striatum after treatment with 3-NP (our results) or MPTP in mice [18], or after brain ischemia in rats [17]. What could be the reason for the reduced mortality we observed? A first possibility is hormesis, a well established phenomenon characterized by increased stress resistance due to the presence of chronic low level of cellular stress [31], [32]. In this case, the subclinical chronic stress could be a consequence of torsinA(ΔE) expression. Analysis of the resting redox potential and products of oxidative metabolism in central neurons of DYT1 KI mice would help determine the influence of torsinA(ΔE) expression on those parameters. Another approach to test this possibility would be to express torsinA(ΔE) acutely in the rodent brain through an inducible system or employing neurotropic viruses. If this protein truly causes oxidative stress, the administration of 3-NP to mice acutely overexpressing torsinA(ΔE) should exacerbate toxicity. Other possible explanation for this unexpected finding is that torsinA could participate in the pathogenic cascade triggered by 3-NP, and this could be impaired by the DYT1 mutation. For instance, in addition to energy depletion and the accumulation of free radicals, 3-NP causes excitotoxicity mediated by striatal dopamine release. Impairment of dopamine release has been observed in various mouse models of DYT1 [33], [34], [35]. Therefore, this defect could contribute to the reduced toxicity observed in DYT1 KI mice. Interestingly, H_2_O_2_, a byproduct of oxidative metabolism, is an endogenous regulator of dopamine release in the striatum [36], potentially linking torsinA function, oxidative metabolism in the ER and dopamine release. It would be interesting to determine the consequences of administering other mitochondrial toxins, such as MPTP or rotenone, to this and other animal models of DYT1 dystonia. If those studies support the protective effect of the DYT1 mutation against mitochondrial dysfunction, additional experiments should be pursued to elucidate the biological mechanism underlying this phenomenon.

Finally, our data supports the upregulation of torsinA expression by 3-NP (Figure 5), as previously shown for MPTP. However, it should be noted that our data derives from western blotting, a semi-quantitative method at best. Nevertheless, we found a significant effect of low dose 3-NP on torsinA levels and a non-significant trend in the high dose cohort. Interestingly, these experiments suggest a higher torsinA upregulation by 3-NP in WT than DYT1 KI mice. If true, this could be a result of the rapid and efficient clearance of torsinA(ΔE) by the ubiquitin proteasome system, offsetting any increases in mRNA levels by 3-NP. Another possibility is that torsinA(ΔE) protects neurons against 3-NP toxicity (as suggested by the mortality data), leading to a lower increment in its levels with the higher 3-NP dose.

In summary, the work presented here demonstrates that the DYT1 mutation does not influence the motor consequences of acquired defects in mitochondrial respiratory chain *in vivo*. Thus, they are unlikely to represent a modifier of DYT1 penetrance or phenotypes.
